# Editorial “pituitary apoplexy—are visual deficits the only indication for emergent surgical intervention?”

**DOI:** 10.1007/s00701-022-05120-1

**Published:** 2022-01-24

**Authors:** Joachim Oertel, Fritz Teping

**Affiliations:** Department of Neurosurgery, Saarland University Medical Centre, Kirrbergerstraße, Building 90.5, 66421 Homburg, Germany

With 2–3%, the initial haemorrhagic presentation of patients with pituitary adenomas is a rare finding [2]. Khawari and colleagues present interesting data of a retrospective multicentre cohort study on management, clinical and oncologic outcomes in such cases. In this study, patients with immediate surgical intervention were compared to those who underwent surgery within three months and with only conservative treatment. Subgroup analysis revealed comparable outcomes for all groups regarding visual and endocrinological findings. Interestingly, patients with subacute surgical therapy within three months showed higher rates of further oncological treatment — but without reaching statistical significance. However, 76% of the patients treated conservatively only did not require any additional treatment at all. Consequently, the authors suggest decision-making on the treatment modality based on visual symptoms rather than oncological aspects.

Surgery in the acute phase is mainly aimed at an early decompression of the visual apparatus or cranial nerves [1]. Reported outcomes prove an effective recovery of up to 94% of visual symptoms depending on surgical timing [3, 8, 9]. Endocrinological improvement is less pronounced with partial recovery rates of up to 23% [7, 8]. However, in our opinion, devastating headaches — as the main symptom of pituitary apoplexy — tend to move into the background in scientific reports. This may be attributed to the limited prospective long-term relevance for the patient. However, in our experience, early surgery is the most effective strategy for pain relief in these patients. Regarding low surgery-related complication rates in experienced high-volume centres, surgical treatment for pain resolution also appears as an essential indication.

Oncologic control somewhat resembles a secondary goal of emergent surgery. The presented study shows that the percentage of patients requiring further oncologic treatment is similar in the surgical versus non-surgical group. This aspect emphasises that the indication setting for emergent surgery depends somewhat on clinical factors than oncologic control. However, a simultaneous tumour resection would be favourable if acute surgery is conducted for visual or pain symptom relief. In this context, endoscopic surgery can be beneficial due to reliable anatomical identifications in high-definition visualisation while preserving endocrinological function [4–6].

As recognised by the authors, the heterogeneous treatment strategies and case-based decisions limit the scientific evaluation. However, the presented data should be considered an accurate reflection of current practice. On the one hand, the presented results underline the effectiveness of such individual treatment decisions. On the other hand, since prospective, randomised investigations are challenging, current recommendations are mainly based on expert opinions. Until the relevant data is available, our recommendation for emergent surgical intervention includes the presence of refractory headaches, especially in combination with visual deficits and endocrinological dysregulation.
